# Comparative Effects of *R-* and *S-*equol and Implication of Transactivation Functions (AF-1 and AF-2) in Estrogen Receptor-Induced Transcriptional Activity 

**DOI:** 10.3390/nu2030340

**Published:** 2010-03-15

**Authors:** Svitlana Shinkaruk, Charlotte Carreau, Gilles Flouriot, Catherine Bennetau-Pelissero, Mylène Potier

**Affiliations:** 1 ENITA de Bordeaux, Unité Micronutriments Reproduction Santé, 1 Cours du Général de Gaulle, CS 40201, F-33175 Gradignan cedex, France; Email: s-poix-shinkaruk@enitab.fr (S.S); chaqx@hotmail.com (C.C.); c-bennetau@enitab.fr (C.B.-P.);; 2 CNRS UMR 6026, 35042, Equipe "Récepteur des œstrogènes et destinée cellulaire", Rennes cedex, France; Email: gilles.flouriot@univ-rennes1.fr

**Keywords:** equol, enantiomeric forms, estrogen receptor, transactivation function, phytoestrogens

## Abstract

Equol, one of the main metabolites of daidzein, is a chiral compound with pleiotropic effects on cellular signaling. This property may induce activation/inhibition of the estrogen receptors (ER) a or b, and therefore, explain the beneficial/deleterious effects of equol on estrogen-dependent diseases. With its asymmetric centre at position C-3, equol can exist in two enantiomeric forms (*R*- and *S*-equol). To elucidate the yet unclear mechanisms of ER activation/inhibition by equol, we performed a comprehensive analysis of ERa and ERb transactivation by racemic equol, as well as by enantiomerically pure forms. Racemic equol was prepared by catalytic hydrogenation from daidzein and separated into enantiomers by chiral HPLC. The configuration assignment was performed by optical rotatory power measurements. The ER-induced transactivation by *R*- and *S*-equol (0.1–10 µM) and 17b-estradiol (E2, 10 nM) was studied using transient transfections of ERα and ERβ in CHO, HepG2 and HeLa cell lines. *R-* and *S-*equol induce ER transactivation in an opposite fashion according to the cellular context. *R-*equol and *S-*equol are more potent in inducing ERα in an AF-2 and AF-1 permissive cell line, respectively. Involvement of ERα transactivation functions (AF-1 and AF-2) in these effects has been examined. Both AF-1 and AF-2 are involved in racemic equol, *R-*equol and *S-*equol induced ERα transcriptional activity. These results could be of interest to find a specific ligand modulating ER transactivation and could contribute to explaining the diversity of equol actions *in vivo*.

## Abbreviations

AF, transactivation function; DCM: dichloromethane; DMEM, Dulbecco’s modified Eagle medium; E2, 17β-estradiol; equol, equol; ER, estrogen receptor; FCS, fetal calf serum; HRT, hormone replacement therapy; ICI, ICI 182,780; IPA: isopropyl alcohol; SEM, standard error to the mean; SERM, selective estrogen receptor modulator; TFA: trifluoroacetic acid; V, vehicle.

## 1. Introduction

Estrogens are used in hormonal replacement therapy (HRT) to prevent menopausal symptoms such as hot flushes, urogenital atrophy, but also osteoporosis in postmenopausal women. Unfortunately, HRT has not lived up to its potential to improve health in women. Estrogens have been associated with an increased incidence of breast and endometrial cancers, which has led to the use of antiestrogens and selective estrogen receptor modulators (SERM) such as tamoxifen and raloxifen, which exhibit a safer profile. However, since undesirable effects persist, numerous investigators continue to search for better SERM for HRT. Much research has been conducted into the health benefits of consuming soy foods, with soy isoflavones and soy protein being implicated as protective against a variety of diseases including heart and vascular diseases, osteoporosis and hormone-dependant cancers (such as those of the breast and prostate) [1,2,3]. Despite their popularity and putative health benefits, it is clear that we need to know much more about the molecular mechanisms, safety and efficacy of these compounds as natural SERM, before they can be recommended to postmenopausal women either as pharmaceutical or nutraceutical agents or as food additives.

Equol [7-hydroxy-3-(4’-hydroxyphenyl)-chroman], is a non-steroidal estrogenic compound found in high concentrations in the urine of about 40% of the adults consuming soy foods [4]. Equol, beside o-desmethyl angolensin, is produced by the colonic bacterial biotransformation of the soy isoflavones aglycone daidzein [5,6,7,8]. Equol is a chiral molecule with an asymmetric centre at position C-3 and can occur in two enantiomeric forms, *R-* and *S-*equol. The absolute configuration of natural (-)-equol, produced by the intestinal bacterial flora, was assigned to *S-*configuration [9,10].

After the identification of equol in biological liquids, the total synthesis was of great importance in order to confirm the chemical structure, and then to provide a sufficient amount of this compound for biological activity studies. Racemic (±)-equol can be synthesized from daidzein and formonetin, which are readily available in sufficient quantities from plants or can be prepared by chemical synthesis. The key transformation step involves the reduction of a vinylogous ester to an ether group. The method most often used during the last years, was the hydrogenation of daidzein by hydrogen in acetic acid with 10% palladium on carbon as a catalyst [11,12]. Recently, transfer hydrogenation was proposed as an alternative to classic hydrogenation, and different catalysts were tested [13]. Paerlman’s catalyst (20% Pd(OH)_2_) was found to be highly effective in the reduction of formonetin, daidzein [13] and corresponding isoflavene [14]. A “biomimetic” reduction of formonetin with dihydroacridine as a hydride donor was also proposed [13]. Recently described, a new original synthetic approach to racemic equol provided a direct construction of the isoflavan skeleton via a Diels-Alder reaction of *o*-quinone methides [15].

The pure *S*-equol enantiomer can be produced by microbiological methods [16,17]. The first total synthesis of *S*-equol was described only three years ago. This approach, based on an Evan alkylation and an intramolecular Bichwald etherification, needed the use of organolithium reagents for the alkylation step, which could not be improved and gave modest yield (9.8% of overall yield) [18]. A new alternative route employed allylic substitution and afforded the *S*-isomer in 24.6% yield over 13 steps [19]. Both *R*- and *S*-equol of high stereoselective purity have been prepared by enantioselective hydrogenation of *O*-protected chromene in the presence of an Ir catalyst having a chiral ligand [20]. Therefore, all synthetic approaches to pure enantiomeric forms remain still expensive and time consuming. The semi-preparative chiral-phase HPLC provides ready and relatively rapid access to both *S*- and *R*-equol in quantity sufficient for *in vitro* studies [13,17,21,22].

Most of published results on the biological activities of equol *in vitro* are available for the racemate, with the exception of Magee *et al.* [22], who evaluated the effects of racemic equol and *S-*equol on breast and prostate cancer cell lines. Their main findings were that racemic and *S*-equol show equipotent biological effects on proliferation and invasion of these cell lines, while the compounds have different abilities to protect against induced DNA damage [22].

Equol is strikingly similar in chemical structure to estrogens and is therefore capable of binding weakly to estrogen receptors (ER) [23]. The effects of 17β-estradiol (E2) and related compounds, such as non-steroidal estrogens and equol, are mediated by two members of the nuclear receptor superfamily, ERα and ERβ, which are coded by separate genes. ER use two transactivation functions (AF), located in their N-terminal (AF-1) and C-terminal (AF-2) domains. Once activated by ligand binding, these AF recruit co-regulators of gene transcription. The transcriptional activity of the AF-2 region is dependent on ligand binding, while AF-1 is constitutively active when isolated. The transcriptional activity of ERα can be promoted through functional cooperation between both AF-1 and AF-2 or through each AF acting independently [24]. 

Therefore, different forms of equol may produce clinical and/or experimental effects distinct from estrogens by differentially triggering ER transcriptional activity. To test this hypothesis, we have first, prepared the pure enantiomeric forms of equol, using semi-preparative chiral phase HPLC, in order to compare the effects of racemic equol and *R-* and *S-* enantiomers on the transcriptional activity of ERα and ERβ. Furthermore, the present study investigated the roles of the AF domains, and more particularly of the AF-1 domain, in the ability of *R-* and *S-*equol to induce ERα transactivation. For these purposes, transient transfections of ERα constructs were performed in ER-negative CHO, HeLa and HepG2 cell lines. 

## 2. Results and Discussion

### 2.1. Chemical Synthesis and Chiral Separation

**Figure 1:**
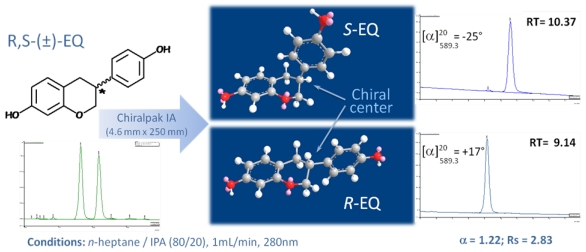
Separation of equol enantiomers on Chiralpak® IA stationary phase.

In this study, the racemic equol was prepared by catalytic hydrogenation from daidzein, which was synthesized as previously described [25]. *R*- and *S*-equol were then separated using a new chiral stationary phase Chiralpak® IA [26]. Different mobile phases were investigated (Table 1). The shorter retention times should be considered and a compromise between different chromatographic parameters should be found for providing an efficient semi-preparative separation. It was found that the mixture *n*-heptane-isopropanol (*n*-Heptane/IPA (80/20, V/V)) had the highest potential in terms of enantioselective separation of equol with the retention of enantiomers in an appropriate time range (Figure 1). Loading studies have been run and scaled up to 10 mm diameter column. Good separation can be achieved with loading up to 7 mg of the racemate per injection. The best loading found in literature data was around 3 mg per injection [16].

**Table 1:** Examples of enantiomeric separations of racemic equol on chiral stationary phase Chiralpak® IA using different mobile phases.

Eluent	Flow rate, mL/min	RT1	RT2	α	Rs
MeOH/TFA (99.9/0.9)	0.5^a^	18.59	20.32	1.11	1.27
*n*-Heptane/EtOAc/TFA (85/15/0.1)	1.0^a^	34.56	40.13	1.18	2.58
*n*-Heptane/DCM/IPA/TFA (50/47.5/2.5/0.1)	1.0^a^	27.94	32.04	1.17	3.09
*n*-Heptane/IPA (80/20)	1.0^a^	9.14	10.37	1.22	2.83
*n*-Heptane/IPA (80/20)	3.0^b^	13.41	15.17	1.17	3.92

^a^ analytical column; ^b^ semi-preparative column. RT: retention time; a: selectivity, or separation factor; Rs: resolution; TFA: trifluoroacetic acid; DCM: dichloromethane; IPA: isopropyl alcohol.

The optical rotations obtained from the isolated peaks 1 and 2 in methanol have positive ([α]^20^_589.3_ = +17°) and negative ([α]^20^_589.3_ = −25°) values, respectively. Since *S*-equol was reported to have a negative specific rotation ([α]^20^_589.3_ = −23.5° in ethanol [18]), the order of elution was assigned as *R*-equol (peak 1) and then *S*-equol (peak 2). The enantiomeric purity of *R*- and *S*-equol were +99% and 97.5%, respectively. 

### 2.2. Transcriptional Activation of ER by Different Enantiomeric Forms of Equol

**Figure 2:**
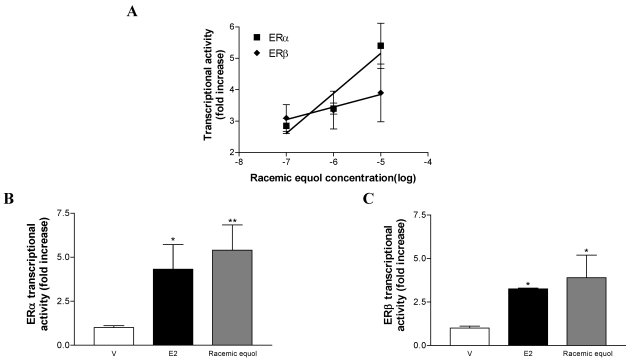
Transcriptional activation of ER by racemic equol in CHO cells. The effects of 0.1, 1 and 10 µM of racemic equol on ERα and ERβ transcriptional activities (**A**) and the effects of control (Vehicle (V), EtOH 0.01%), E2 (10 nM) and racemic equol (10 µM) on ERα (**B**) and ERβ (**C**) transcriptional activities. Cells were co-transfected with ERα or ERβ and the ERE-TK-LUC. Data are expressed as reporter fold induction compared to control. Shown are means ± SEM of 6–10 independent experiments, each performed in triplicate. Comparisons performed between experimental conditions are described in “Experimental Section”. Statistical significances are indicated by * or ** for comparison to control for p < 0.05 or p < 0.01, respectively.

In CHO cells, racemic equol (0.1 µM to 10 µM) induces ERα and ERβ transactivation on the ERE-TK-LUC reporter gene in a dose-dependent fashion as calculated by linear regression (R^2^ = 0.90 for ERα and 0.95 for ERβ), as shown in Figure 2A. For ERα, a significant increase in transcriptional activity is obtained with 1 and 10 µM of racemic equol (3.4 ± 0.4-; 5.4 ± 1.4-fold increase compared to control, p < 0.05 and p < 0.01, respectively). For ERβ, only the highest concentration of racemic equol (10 µM) induces a significant effect (3.9 ± 1.3 fold increase compared to control, p < 0.05). Racemic equol (10 µM) induces a significant ERα and ERβ transactivation similar to the one induced by 10 nM 17b-estradiol (E2), the endogenous ligand of ER. The ERβ−induced transactivation by E2 and racemic equol in CHO cells is slightly lower than that of ERα (Figure 2B and C). E2 and equol effects on both ERα and β are completely abolished by treatment with 1 µM ICI (data not shown).

We observe that similar effects to E2 are typically achieved at concentrations that are three-orders of magnitude higher, which can be reached physiologically with a soy-rich diet. This is in accordance with the fact that the relative binding affinity of racemic *R-* and *S-*equol measured on recombinant ER, is generally in the order of 100 to 1000-times less than that of E2 [13]. 

Serum concentrations of equol are quite different between women (with an equol-producer status) from various geographic areas and/or specific diets. We have shown that serum concentrations of equol reach 0.6 µM following consumption of soy supplements and up to 3 µM after ingestion of 50 mg total isoflavones (about 30% daidzin) twice a day [27,28]. A recent study demonstrated a high equol bioavailability, with racemic, *R-* and *S-*equol concentrations in plasma from 0.4 up to 2 µM after a single bolus administration of equol (20 mg) [29]. 

We have previously demonstrated that the ability of phytoestrogens, such as genistein, daidzein and racemic equol to act as ER agonists is independent of the cellular context (AF-1 or AF-2 permissive) [30]. Therefore, it was of particular interest to determine the mechanisms of action of enantiomeric forms of equol on ER transcriptional activation in epithelial cell lines, which have different AF permissiveness. 

In HepG2 cells, racemic equol, *R-*equol and *S-*equol (0.1 µM–10 µM) induce ERα and ERβ transactivation on the ERE-TK-LUC reporter gene in a dose-dependent fashion, as calculated by linear regression (R^2^ = 0.85; 0.95; 0.95 for ERα and 0.90; 0.94; 0.78 for ERβ, respectively, data not shown).

**Figure 3:**
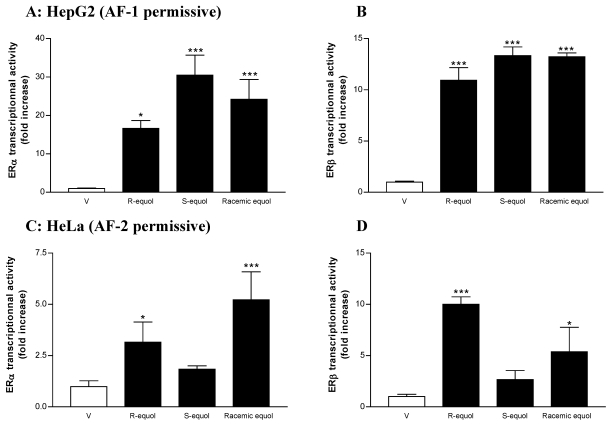
Transcriptional activation of ER by enantiomeric forms of equol in HepG2 (AF-1 permissive) and HeLa (AF-2 permissive) epithelial cells. The effects of control (Vehicle (V), EtOH 0.01%), *R-* and *S-*forms and racemic equol (10 µM) on ERα (**A, C**) and ERβ (**B, D**) transcriptional activities in HepG2 and HeLa cell lines, respectively. Cells were co-transfected with ERα or ERβ and the ERE-TK-LUC. Data are expressed as reporter fold induction compared to control. Shown are means ± SEM of 2–5 independent experiments, each performed in triplicate. Comparisons between experimental conditions are described in “Experimental section”. Statistical significances are indicated by * or *** for comparison to control for p < 0.05 or p < 0.001, respectively.

Racemic equol, *R-*equol and *S-*equol (10 µM) induce a significant ERα and ERβ transcriptional activation (24.2 ± 5.2; 13.2 ± 0.4, 16.6 ± 2.1; and 10.9 ± 1.2; 30.5 ± 5.2; 13.3 ± 0.8 for ERα and ERβ, respectively) compared to control, as shown in Figure 3A and B. In HepG2 cells, *S-*equol induces a higher ERα transcriptional activation than *R-*equol (p < 0.05). ERα transcriptional activation induced by *R-*equol (10 µM) is not different from racemic equol. The ERβ-induced transactivation by *R-*equol, *S*-equol and racemic equol (10 µM) in HepG2 cells is overall lower than that of ERα (Figure 3A and B). There is no difference between racemic equol, *R-*equol and *S*-equol (10 µM) ERβ−induced transactivations in HepG2 cells. E2, racemic equol, *R-* and *S-*equol effects on both ERα and ERβ are completely abolished by treatment with 1 µM ICI (data not shown). 

In HeLa cells, racemic equol and *R-*equol (0.1 µM-10 µM) induce ERα and ERβ transactivation on the ERE-TK-LUC reporter gene in a dose-dependent fashion, as calculated by linear regression (R^2^ = 0.72; 0.78 for ERα and 0.99; 0.78 for ERβ, respectively, data not shown). In contrast, *S-*equol even at the highest concentration tested (10 µM) does not induce significant ER transcriptional activation (1.8 ± 0.2 and 2.7 ± 0.9 for ERα and ERβ, respectively) compared to control (Figure 3C and D). As for CHO cells (Figure 2), the ERα and ERβ−induced transactivations by racemic equol (10 µM) in HeLa cells are similar (5.2 ± 1.4 and 5.4 ± 2.4 fold increase compared to control, p < 0.001 and p < 0.05, respectively). E2, racemic equol, *R-* and *S-*equol effects on both ERα and ERβ are completely abolished by treatment with 1 µM ICI (data not shown). In HeLa cells, both ERα and ERβ transcriptional activations induced by *R-*equol (10 µM) is not different from racemic equol. *R-*equol induces a stronger ERβ transcriptional activation than *S-*equol (p < 0.01). Interestingly, the highest concentration of *R-*equol (10 µM) induces a stronger ERβ transcriptional activation than ERα (10.0 ± 0.7 and 2.9 ± 1.0 fold increase compared to control, p < 0.001 and p < 0.05, respectively).

Taken together, our results clearly demonstrate that *R-*equol and *S-*equol induce ERα and ERβ transactivations in a different manner in regards to the AF permissiveness of the cell line. While *S-*equol is more potent to induce ERα in the AF-1 (HepG2) permissive cell line, *R-*equol appears to be more effective in inducing ERα in the AF-2 (HeLa) permissive cell line. 

Several studies have evaluated the ER subtypes binding affinity and/or transcriptional activity of racemic equol [10,31,32,33] and equol enantiomers [13]. Taken together these authors report that (1) in binding assays, equol has a distinctively higher binding affinity, but only slight preference for transactivation of ERβ compared to ERα; (2) *S–*equol has a high binding affinity preference for ERβ, while *R-*equol binds more weakly and with a preference for ERα; (3) racemic, *R-* and *S-*equol are ER agonists in transcriptional activity studies, and (4) in contrast to the slight ERα preference of *R-*equol, *S-*equol has no ER subtypes preference in terms of transcriptional potency [13].

It is well known that the estrogenic potency of compounds is a complicated phenomenon, which results from a number of factors, such as the nature of the inducer, including antiestrogens, xenoestrogens and phytoestrogens, but also the differential effects on the transactivation functionalities of the receptor, the particular co-activators recruited, the cell- and target promoter-contexts, the relative expression of each ER subtype [34,35,36,37,38,39] and the cell differentiation stage [34,37,40,41]. Therefore, the use of different model to study transcriptional potencies (HEC-1 cell line and an (ERE)_2_-PS2-LUC reporter gene [13] *versus* HeLa and HepG2 cell lines and (ERE)-TK-LUC reporter gene, for instance, may also explain this discrepancy. 

The use of ERα constructs expressed in ER-negative backgrounds has been a powerful technique for studying the function of various domains in the ER [37,40,41]. To further examine the role of AF-1 in the ERα-induced transactivation by *R-* and *S-*equol, we used expression vectors of full length ERα (ERα) or truncated ERα in the A/B domain (ERαΔAF-1) in both epithelial cell lines. We compared the transcriptional efficiency of both ERα constructs on estrogen sensitive reporter genes in HepG2 (AF-1 permissive) and HeLa (AF-2 permissive) epithelial cell lines. Similar expression of the different ERα variants was controlled by Western blots as previously described [42]. 

**Figure 4:**
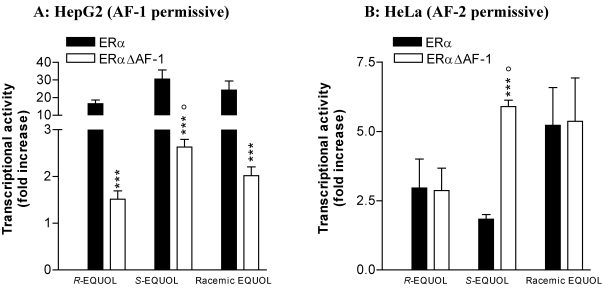
Transcriptional activation of ERα and ERαΔAF-1 by enantiomeric forms of equol in HepG2 (AF-1 permissive) and HeLa (AF-2 permissive) epithelial cells. The effects of *R-* and *S-*forms and racemic equol (10 µM) on ERα and ERΔAF-1 transcriptional activities in HepG2 (**A**) and Hela (**B**) cell lines, respectively. Cells were co-transfected with ERα or ER ΔAF-1 and the ERE-TK-LUC. Data are expressed as reporter fold induction compared to control (Vehicle (V), EtOH 0.01%). Shown are means ± SEM of 2-5 independent experiments, each performed in triplicate. Comparisons between experimental conditions were performed as described in methods section. Statistical significance for comparison between ER constructs or between *R*- and *S*-equol is indicated by *** for p < 0.001 or by ° for p < 0.05, respectively.

In HepG2 cells, where ERα transactivation is mainly ensured by AF-1 (ERα >> ERαΔAF-1), racemic, *R-*and *S-* equol (10 µM) induce, as expected, a higher transactivation of ERα than ERαΔAF-1 (Figure 4A). *S-*equol induces a higher ERαΔAF-1 transcriptional activation than *R-*equol (p < 0.05) in AF-1 permissive cells. In HeLa cells, ERα transactivation is mainly ensured by AF-2 (ERα ≈ ERαΔAF-1). Racemic equol and *R-*equol (10 µM) induce a similar transactivation of both ERα and ERαΔAF-1 (Figure 4B), indicating that deletion of AF-1 has no effect on ERα-induced transactivation for both compounds. Similar results are obtained in CHO cells (data not shown) with racemic equol presenting a similar transactivation profile as E2, as previously described [30]. In contrast, *S-*equol does not induce similar transcriptional activation of ERα and ERαΔAF-1 in HeLa cells. Deletion of AF-1 enhances ERα transcriptional activity to reach the level of the one induced by racemic equol and significantly different than *R-*equol (p < 0.05, Figure 4B). This result indicates that this property of *S-*equol to induce ERα transcriptional activation through AF-2 is partly repressed by AF-1. This could be due to a conformational change of the ligand binding domain, which inhibits, at least partially, the activity of AF-1 in HeLa cells [24]. ICI (1 µM) completely blocked the effects of E2 and enantiomeric forms of equol in both cell lines (data not shown). *R-* and *S-*equol are therefore capable of inducing ERα transactivation through activation of both AF, however, *S-*equol effect through AF-2 may be repressed by AF-1. 

Taken together, our results indicate that while *R-*equol and *S-*equol are more potent in inducing ERα transactivation in an AF-2 and in an AF-1 permissive cell line, respectively, both compounds *R-*equol and *S-*equol are capable of inducing ERα transactivation through activation of both AF-1 and AF-2 domains. However, in contrast to *R-*equol, *S-*equol-induced ERα transcriptional activation through AF-2 is (1) higher in the AF-1 permissive cell line, and (2) partly repressed by AF-1 in the AF-2 permissive cell line.

We conclude that racemic, *R-* and *S-*equol exert distinct effects on ER transcriptional activity, which cannot be solely explained by their differential affinities for the ER subtypes [13], but may be due to the differential effects on the transactivation functionalities of the receptor and the cell differentiation stage [30,34,37,40,41,42]. Given the pleiotropic actions of phytoestrogenic compounds, it is possible that they are also affecting other biological processes in addition to these mechanisms, such as cellular metabolism, relative expression of each ER subtype, non-genomic activities of ER or other signal transduction pathways (such as MAPK). Further *in vitro* studies are therefore needed to elucidate the pathways involved in equol effects.

Approximately 40% of Humans have the bacteria capable of producing equol. The ability to produce equol following ingestion of soy isoflavones is of particular interest, because it has been demonstrated *in vitro* and in some animal models that equol is more biologically active than its precursor daidzein and the alternate metabolite, o-desmethyl angolensin [5,6,7,8]. More importantly, studies report relationships between the equol-producer phenotype and reduced risk factors for several chronic diseases and differential responses to interventions (for review, [7,43]). Given that it is exclusively the *S-*equol enantiomer that is produced *in vivo* by the gut microflora, [9,10], our findings may have implications regarding the effects of equol *in vivo*. In particular, since ERα activity is mediated through AF-1 in differentiated cells and through AF-2 in well-dedifferentiated cells [41], *S-*equol may differentially modulate processes involving ERα activation, such as cell differentiation and/or proliferation, as in breast cancer.

In this regard, a very recent study demonstrated, in a rat model of chemically-induced-tumors, that *S-*equol has no chemopreventive action *in vivo*, while the unnatural enantiomer *R-*equol was potently chemopreventive [44]. It is clear that further studies are needed to elucidate specifically the biological (for example, antigenotoxic and/or antioxidant properties) of such compound. However, these results may contribute to explain the diversity of daidzein and/or equol actions *in vivo* and particularly as *S-*equol, as food supplement, is being clinically developed for the treatment of prevention of menopausal symptoms [45].

## 3. Experimental Section

### 3.1. Chemicals and Instruments

All chemical reagents and solvents were purchased from Sigma Aldrich Chemical Co. (Saint Quentin Fallavier, France), Alfa Aesar. (Bisheim, France), or Acros Organics France (Noisy, France). 17β-estradiol (E2) was purchased from Sigma Aldrich Chemical Co. (Saint Quentin Fallavier, France) and ICI 182,780, (ICI) from Tocris (United Kingdom). For cell culture, DMEM culture medium and fetal calf serum (FCS) were from Sigma Aldrich Chemical Co. (Saint Quentin Fallavier, France) while antibiotics and DMEM/F12 phenol red free medium were purchased from Invitrogen (France). FuGENE^TM^ 6 for transient transfections was provided by Roche Diagnostics (France).

Melting points were determined with a Stuart Scientific melting point apparatus SMP3 and are uncorrected. ^1^H and ^13^C NMR spectra were recorded with a Bruker AC-300 FT (^1^H: 300 MHz, ^13^C: 75 MHz). The chemical shifts (δ) and coupling constants (J) are expressed in ppm and Hz, respectively. Optical rotations were measured on a Bellingham Stanley Polarimeter ADP220 at ambient temperature. Thin-layer chromatography (TLC) was performed using SDS TLC plates, 0.25 mm, particle size 15 μm, pore size 60 Å. Merck silica gel 60 (70–230 mesh) and (0.063–0.200 mm) were used for flash chromatography. The spots were visualized with a UV lamp.

### 3.2. Chemical Synthesis of (±)-Equol

Palladium-on-charcoal (10%, 0.5g) was added to a well-stirred solution of daidzein (2 g, 7.9 mmol) in 95% ethanol (200 mL). The mixture was degassed and placed under hydrogen atmosphere at room temperature and atmospheric pressure for 24 h. After filtration and evaporation of the solvent, the crude product was recrystallized from ethanol/water to afford the target product as white crystals (1.2 g, yield 82%, purity ≥ 97%, m.p. 156–156.5 °C (lit. 158 °C [11])). 

*^1^H NMR (acetone-d6, 300 MHz):* δ 2.84 (m, 2H, H-4), 3.11 (m, 1H, H-3), 3.93 (dd, ^2^*J_2ax-2eq_* = ^3^*J _2ax-3_* = 10.5 Hz, 1H, H-2_ax_), 4.19 (ddd, ^2^*J_2ax-2eq_* = 10.5 Hz, ^3^*J_2eq-3_* = 3.6, ^4^*J_2eq-4ax_* = 1.8, 1H, H-2_eq_), 6.30. (d, ^4^*J* = 2.3 Hz, 1H, Har-8), 6.37 (dd, ^3^*J* = 8.3 Hz, ^4^*J* = 2.3 Hz, 1H, Har-6), 6.83 (d, ^3^*J* = 8.6 Hz, 2H, Har-3’,5’), 6.90 (d, ^3^*J* = 8.3 Hz, 1H, Har-5), 7.16 (d, ^3^*J* = 8.6 Hz, 2H, Har-2’,6’), 8.19 (br s, 1H, OH), 8.31 (br s, 1H, OH).

*^13^C NMR (acetone-d6, 75 MHz):* δ 156.7 (CQ-4’), 156.3 (CQ-9), 155.1 (CQ-7), 132.5 (CQ-1’), 130.1 (CHar-5), 128.3 (CHar-2’,6’), 115.4 (CHar-3’5’), 113.2 (CQ-10), 107.9 (CHar-6), 102.7 (CHar-8), 70.7 (CH_2_-2), 37.9 (CH-3), 31.8 (CH_2_-4).

### 3.3. Chromatographic Resolution of R- and S- equol.

Enantiomeric separation was performed on a Varian Prostar chromatographic system with UV detection at a wavelength of 280 nm. A Daicel Chiralpack^®^ IA column [26] with amylase tris (3,5-dimethylphenylcarbamate chiral phase immobilized on 5 µm silica-gel (analytical column 250 × 4.6 mm, semi-preparative column 250 × 10 mm, Chiral Technologie Europe, Illkirch, France) with a Chiralpak^®^ IA guard column were used. The mobile phase selected for the method consisted of a mixture of *n*-heptane/IPA (80/20, v/v) delivered in isocratic elution mode. The flow rates were 1 mL/min and 3 mL/min for analytical and semi-preparative columns, respectively. For semi-preparative separation, the injection volume was 100 µL of equol solution in IPA (50 mg/mL). The elution order and retention times were as follows: RT 9.14 min for *R*-equol and RT 10.37 min for *S*-equol.

### 3.4. Plasmids

Expression vectors pSG5, pSG human (h)ERα, pSGERαΔAF-1 and pCMV-β galactosidase as well as ERE-TK-LUC reporter genes were previously described [42]. Expression vector for human (h)ERβ (pSG5ERβ) was provided by Pr. J.A. Gustafsson’s department (Dpt Biosciences and Medical Nutrition, Karolinska Institute, Sweden).

### 3.5. Cell Culture and Transient Transfection Experiments

CHO, HeLa and HepG2 cell lines were routinely maintained in DMEM supplemented with 5% FCS and antibiotics. For experimental conditions, phenol red free medium DMEM/F12 supplemented with 2.5% charcoal-stripped FCS was used (Experimental medium).

Transfections were carried out with FuGENE^TM^ 6 as described previously [42] with 50 ng of total DNA consisting of the expression vector, the reporter gene and the pCMV-β galactosidase internal control (10, 20 and 20 ng, respectively). Cells were treated either with different concentrations of E2 and equol (10 nM and 0.1 to 10 µM, respectively), ICI (1 μM) or vehicle (V, 0.01% EtOH), or a combination of these compounds as indicated. Cells were harvested and luciferase and β-galactosidase assays were performed as previously described [42]. The reporter gene activity was obtained after normalization of the luciferase activity with the β-galactosidase activity.

### 3.6. Statistical Analysis

Shown are the means ± SEM of 2 to 10 independent experiments, each performed in triplicate as indicated. One-way ANOVA and Dunnett’s multiple comparison post-hoc test or Student’s t-test were performed for the statistical analysis between experimental conditions (GraphPad Prism^®^, USA). Dose–dependant effects were assessed by linear regression (GraphPad Prism^®^, USA). Statistical significance is indicated by 1, 2, and 3 symbols (* or ◦) corresponding to p < 0.05, p < 0.01 and p < 0.001, respectively.

## 4. Conclusions

Equol, one of the main metabolites of daidzein is being clinically developed as a food supplement to treat estrogen-related diseases. Understanding how natural estrogenic compounds elicit clinical selective effects is key to the development of safer HRT. Equol is a chiral compound, and induction of activation/inhibition of the ER may depend on the enantiomeric form and purity of equol. Catalytic hydrogenation of daidzein followed by chiral HPLC separation provided ready and rapid access to racemic, *R-* and *S-* forms of equol in sufficient quantities, and allowed us to examine their differential effects on the two ER subtypes. Good chiral separation with semi-preparative isolation of more than 3 mg of each enantiomer per injection was achieved using a new immobilized chiral stationary phase. We have shown that high concentrations (10 µM) of *R-*equol and *S-*equol induce ERα and ERβ transcriptional activation differently according to the cellular context. *R-*equol and *S-*equol are more potent to induce ERα in the AF-2 and AF-1 permissive cell lines, respectively. The *S-*enantiomer has little transcriptional potency on both ERα and ERβ in an AF-2 permissive cell line. ERα transcriptional activation by both ennatiomers involves their capacity to act mainly through AF-1 and AF-2. This study confirms that racemic, *R-* and *S-* equol are SERM with estrogenic activities. Therefore, in light of our study of the effects of equol and its enantiomeric forms on the two ER, it would appear prudent to evaluate carefully, *in vivo*, the biological effects of not only the isoflavones, but also their metabolites and their enantiomers. Such investigations would greatly help in evaluating the potential effects of the ingestion of soy isoflavones on human health and disease. 
